# Participatory asset mapping and photovoice interviews to scope cultural and community resources to reduce alcohol harm in Chitwan, Nepal

**DOI:** 10.1177/17579139231180744

**Published:** 2023-06-25

**Authors:** R Dhital, H Yoeli, A Adhikari, NP Luitel, A Nadkarni, E van Teijlingen, J Sin

**Affiliations:** UCL Arts and Sciences Department, University College London, 33-35 Torrington Place, London WC1E 7LA, UK; School of Health and Psychological Sciences, University of London, London, UK; Prime Nepal, Bharatpur, Nepal; Transcultural Psychosocial Organization Nepal, Kathmandu, Nepal; Department of Population Health, London School of Hygiene and Tropical Medicine, London, UK; Addictions Research Group, Sangath, Goa, India; Bournemouth University, Poole, UK; School of Health and Psychological Sciences, University of London, London, UK

**Keywords:** alcohol harm, global public health, Nepal, cultural and community assets, photovoice interviews, participatory asset mapping

## Abstract

**Aims:**

To scope the breadth of existing cultural and community assets and how alcohol drinkers and community health workers perceived them in relation to reducing alcohol-related harm.

**Methods:**

The study was conducted in Chitwan, south-central Nepal, which has considerable alcohol problems. Participatory asset mapping was conducted using field notes, photography, and through engaging with communities to explore how community assets affect alcohol consumption. Semi-structured photovoice interviews were conducted with harmful/hazardous drinkers (AUDIT score 8 to 19) and community health workers. Purposive and snowball sampling were used to recruit participants. During interviews, participants used their photographs to reflect on how community assets influenced alcohol use. Thematic framework analysis was used to analyse the data.

**Results:**

We recruited 12 harmful/hazardous drinkers (3 females) and 6 health workers (2 females). The mean AUDIT score of the former was 12.17 (SD ±2.86). Thematic analysis of the photovoice interviews produced three themes: ‘influences and impact of families and communities’; ‘culture and spirituality’; and ‘nature and the environment’. The community mapping produced five assets that promoted alcohol consumption: (1) availability; (2) advertising; (3) negative attitudes towards users; (4) festivals/gatherings; and (5) illiteracy/poverty. Six assets that discouraged consumption were: (1) legislation restricting use; (2) community organisations; (3) cultural/spiritual sites; (4) healthcare facilities; (5) family and communities; and (6) women’s community groups. Those from certain ethnic groups consumed more alcohol, experienced more family discord, or felt stigmatised due to their drinking. Assets ‘festivals/gatherings’ and ‘negative attitudes toward users’ and the theme ‘family and communities’ concerned with relationships and community activities were perceived to both promote and reduce alcohol use.

**Conclusions:**

This study provides new insight into a variety of cultural and community assets that promote and reduce alcohol use. The study identifies new possibilities to build on visual participatory and arts-based methods that have potential to be effectively implemented at scale.

## Introduction

Alcohol causes around 3.3 million deaths annually, representing 6% of annual global mortality.^1^ Alcohol is one of four main modifiable behavioural risk factors contributing to non-communicable diseases (NCDs);^2^ these risk factors have been recognised in one of the United Nations’ key Sustainable Development Goal (SDG).^3^ In low- and middle-income countries (LMICs) such as Nepal, morbidity and mortality risks are greater per litre of pure alcohol consumed than in higher-income countries.^1,4^ This is largely due to poverty, poor nutrition, adverse living conditions, and poor access to care. These inequities are made worse by the dearth of understanding and availability of the most appropriate and cost-effective approaches to reduce alcohol-related harm in LMICs.

**Figure 1:**
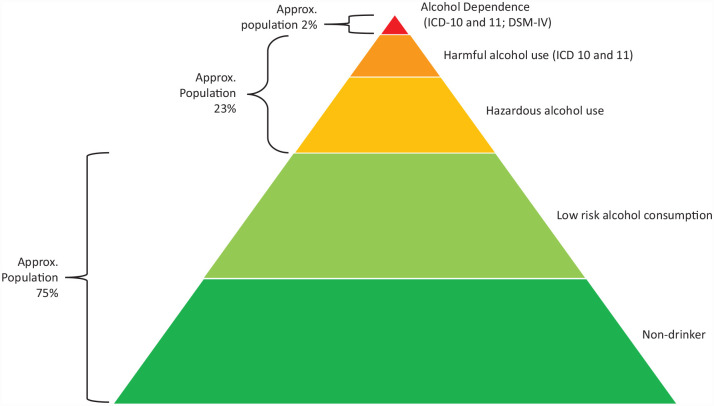
Patterns of alcohol use and disorders (not to scale) Source: Adapted from the study by Saunders *et al*.^5^ Note: *DSM*: Diagnostic and Statistical Manual of Mental Disorders; ICD: International Classification of Diseases.

Figure 1 illustrates the range of alcohol use and disorders associated with patterns of drinking based on data from high-income countries and LMICs.^5^ Where ‘harmful’ use is defined as drinking at levels likely to cause psychological and physical harm, without experiencing full dependence syndromes;^6^ ‘hazardous’ use relates to a pattern of drinking that confers a risk of possible future harm to the user.^6^

In LMICs where health care resource availability is limited, brief psychosocial interventions delivered collaboratively with non-specialist health workers in primary care settings can provide scalable public health options to effectively lower alcohol risk levels and improve health outcomes.^6,7^ Communities from both high-income countries and LMICs have a long social and cultural history with alcohol, including the diverse ways alcohol is produced and consumed. Nepal has a rich cultural heritage where knowledge and practice of the arts, religion, and public health are often interconnected.^8^ These varied experiences and perceptions can be both an asset and a challenge to creating effective ways to reduce alcohol-related harm, especially in communities with a long social and cultural history of alcohol production and consumption. Therefore, designing and delivering brief psychosocial interventions that relate to local cultures and community resources are more likely to be acceptable and relevant to communities. Engaging and culturally sensitive interventions also have better potential to be scaled-up and are more likely to produce effective long-term reductions in alcohol-related harm.

Of the limited alcohol research conducted in LMICs, very few have explored perspectives, contexts, or experiences of individuals and communities.^9,10^ There is also no known intervention study, which has led to significant reduction in alcohol harm in Nepal.^11^ In LMICs’ health systems, resources including healthcare staff are limited. Therefore, alternative approaches are urgently needed to increase availability and access to care by harnessing existing community assets more effectively to promote health and wellbeing.^12^ Such approaches would be framed around a salutogenic paradigm rather than a traditional deficit model. Asset-based alternatives to deficit-based models have been developed from emancipatory approaches to education and community work, often grounded in global alternatives to western medical philosophies.^13^

The use of cultural and community assets in public health can help reduce stigma, raise awareness, and enable engagement of diverse communities.^14,15^ Such public health approaches would require participatory^16^ and co-design^17^ methodologies to examine cultural and community assets, including the arts, cultural sites, natural environment, and community groups on their potential to reduce alcohol harm and associated risks. Therefore, this study aimed to stimulate new thinking on how cultural and community assets could be integrated to co-design alcohol interventions for future evaluation in Nepal and other LMICs. This was achieved through scoping the breadth of cultural and community assets related to alcohol, and exploring attitudes and experiences of alcohol use and existing support to reduce alcohol-related harm.

## Methods

The study had two components: participatory cultural and community asset mapping^18^ sub-study conducted by researchers; and semi-structured photovoice interviews^19^ with local harmful/hazardous drinkers (henceforth ‘harmful drinkers’) and health workers. This article has been prepared following the COREQ (consolidated criteria for reporting qualitative research) standard for qualitative research.^20^

### Setting

Chitwan, a south-central district of Nepal, is an area with considerable mental health and alcohol problems.^21^ Chitwan is a relatively urban district with a population of 719,859; 84% literacy rate (national average = 76%); and a diversity of culture, caste, geography, language, and religion.^22^ The district has government and private hospitals within urban areas, while rural areas comprise of primary health care centres (PHCC) and health posts.^23^ The district offers alcohol detoxification treatment as part of the national Basic Health Service (BHS) Package from government hospitals;^24^ however, the provision of psychosocial interventions to prevent harmful alcohol use is limited. Female Community Health Volunteers (FCHVs) are involved in promoting maternal, child, and public health activities through PHCC government-supported schemes.^25^

### Ethics and study reporting

The study received ethical approval from Nepal Health Research Council (NHRC; Reference-27 /2021 P) and City, University of London (UK; ETH2021-1001).

### The community asset mapping sub-study

Two local researchers (AA and AS) scoped community assets and resources and their relationship with alcohol (January to July 2021). This involved the two researchers observing and photographing a variety of cultural, religious, social, and community venues and activities to explore how these may affect alcohol consumption. Local communities were approached to clarify and gain deeper insight into researchers’ observations. Researchers’ field notes and photographs were discussed regularly within the team during data collection and analysis.

### Semi-structured photovoice interview

#### Recruitment

Purposive and snowball sampling were used by local researchers (AA, PP, and DR) to identify adult (⩾ 18 years), possible harmful drinkers and health workers. The former were defined as scoring 8 to 19 on the Alcohol Use Disorder Identification Test (AUDIT), based on a version validated for use in Nepal.^26,27^ Individuals who scored 20 or more on the AUDIT, an indication of possible dependent drinking, were excluded and advised to seek specialist support for safeguarding reasons and to focus on our study target – harmful drinking population.

Primary health workers and FCHVs were also involved in informing and referring those interested in participating in the study to local researchers. Some FCHVs and primary health workers were aware of or had been involved in the multi-country ‘Programme for Improving Mental Health Care’ (PRIME) research programme for LMICs^28^ (Chitwan was one of the research sites) and received training on how to identify and support those with mental health and alcohol problems. Individuals were informed about the study at PHCCs, health posts, and within social and cultural venues such as community parks or temples. Those who provided informed written consent were recruited to the study.

Primary health workers, referred in this article as ‘health workers’ ranged from basic health assistants, auxiliary health workers, to medical doctors and were recruited by researchers from PHCCs and health posts. These healthcare facilities provide basic services including some mental health support.^23^ Harmful drinker and health worker participants were reimbursed for their travel and received a compensation of Rs 1000 (US$9) for their involvement.

### Semi-structured photovoice interviews data collection

Individual in-depth interviews with 12 harmful drinkers and six health workers were conducted from April to July 2021 either at health care facilities, the Prime Nepal office (local mental health NGO in Chitwan where AA is based), or participant’s home, depending on individual preference. Following informed consent, socio-demographic data were collected from each participant. The semi-structured interview guide was developed following the asset mapping and informed by a review of alcohol research literature from Nepal and other LMICs. The guide was underpinned by theories relating to asset-based models of health.^29^ Interview topics included: general alcohol use; problems associated with drinking; community and cultural assets; and alcohol support and treatment needs of the community. Health workers were asked questions related to their professional experience of working with harmful drinkers and training and support requirements.

Before each interview, all participants were invited to click at least four photographs over a week to either reflect on their experience of alcohol use as a harmful drinker participant, or when engaging with drinkers through their work as a health worker. All participants were also encouraged to explore the possible influence of cultural and community assets on alcohol consumption. All participants were offered a digital camera or a mobile phone by the researcher, or if they preferred, they could use their own device. Throughout the interviews, both harmful drinker and health worker participants were encouraged to refer to their four printed photographs when discussing their experiences and attitudes to alcohol. All participants were asked to reflect on what and how community assets helped or hindered health and wellbeing; and for health worker participants, on ways it affected them when offering support. Each interview lasted between 30 and 75 min, was audio-recorded, transcribed into Nepali, and translated into English for analysis.

### Thematic framework analysis

Field notes and photographs from the community asset mapping were analysed using a thematic framework^30^ to identify factors influencing alcohol use among individuals and communities.

Thematic framework^30^ was also used to analyse the narrative and photographic data. The interviews were first analysed by AA, RD, and HY by re-reading the interview transcripts to familiarise with the content and check for accuracy. Codes were developed inductively from transcripts and photographs as well as deductively from the broad themes covered by the interview guide initially by AA, and further coding were then independently completed by RD and HY. The codes were then collapsed into sub-themes and multifaceted themes through discussion between the wider research team. NVivo20^31^ was used to manage the analysis.

## Results

### Demographics of participants

A total of 18 participants were recruited (see Table 1 for a summary of demographic characteristics). Mean AUDIT score of the 12 harmful drinkers was 12.17 (SD ±2.86, range 9–17). Harmful drinker and health worker and participants were of similar mean age (41 and 44 years, respectively); mostly male (75% versus 67%); were married; and Hindu religion. Half of the harmful drinkers were Brahmins and from other ethnic castes, while health workers were only either from Brahmin (83%) or Chettri (17%) ethnic castes. More health workers had completed college or university education (83%) compared to harmful drinker participants (17%).

**Table 1:** Characteristics of photovoice interviewees.

Characteristics	Harmful/hazardous drinkers (n = 12)	Health workers (n = 6)
Age (years) mean (SD), range	41.25 (SD ±7.67) 29–58	44.50 (SD ±3.94) 39–49
Gender:		
Female	3 (25)	2 (33)
Male	9 (75)	4 (67)
Marital status:		
Married	11 (91.7)	6 (100)
Unmarried	1 (8.3)	/
Religion:		
Hindu	11 (91.7)	6 (100)
Christian	1 (8.3)	/
Ethnicity/caste:		
Brahmin	6 (50.0)	5 (83.3)
Chettri	2 (16.7)	1 (16.7)
Newar	1 (8.3)	/
Dalit or also known as Nepali caste	1 (8.3)	/
Chaudhari	2 (16.7)	/
Education:		
Informal education or literate	3 (25)	1 (16.7)
Secondary school education	7 (58.3)	/
College or university	2 (16.7)	5 (83.3)
Employment:		
Agriculture	5 (41.7)	1 (16.7)
Employed within a service industry	4 (33.3)	5 (83.3)
Self-employed business	2 (16.7)	/
Unemployed	1 (8.3)	/
Number of months of income received during past one year:		
More than 9	8 (66.7)	6 (100)
Between 7 to 9	2 (16.7)	/
Between 3 to 6	2 (16.7)	/

Values are numbers (%) unless stated otherwise. Slash (/) indicates no data were reported by participants for these characteristics.

**Photo 1:**
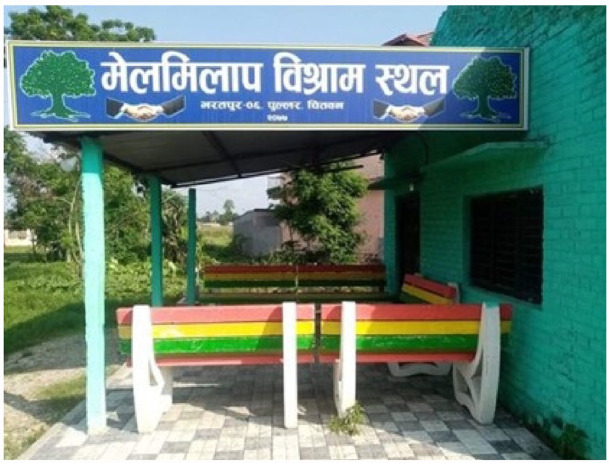
Health Worker, P04, male aged 46 Image of seating area outside a mediation community centre.

**Photo 2:**
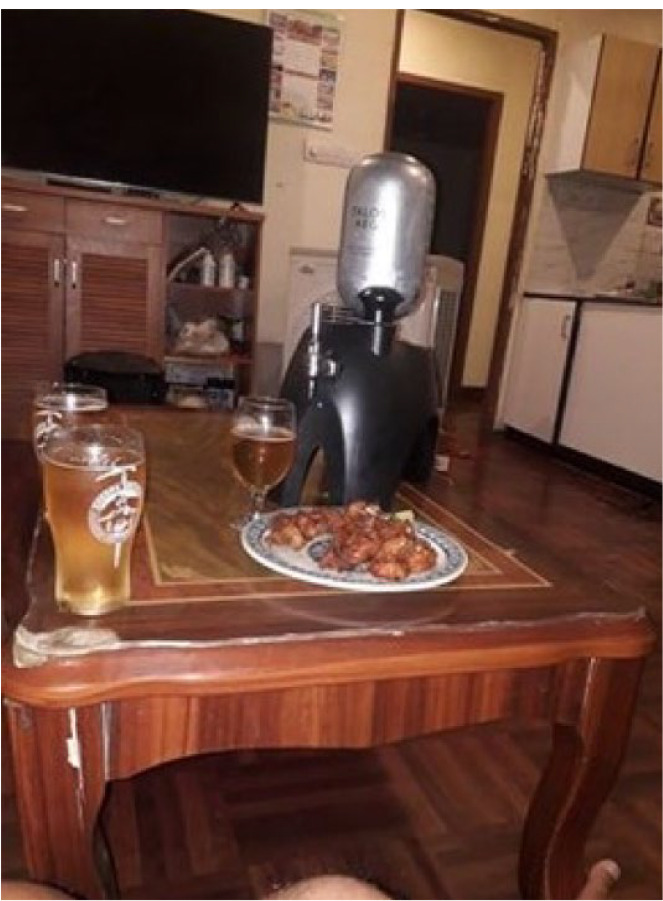
Harmful Drinker, P07, male aged 34 Image of a table with glasses of alcoholic drinks and a plate of food.

**Photo 3:**
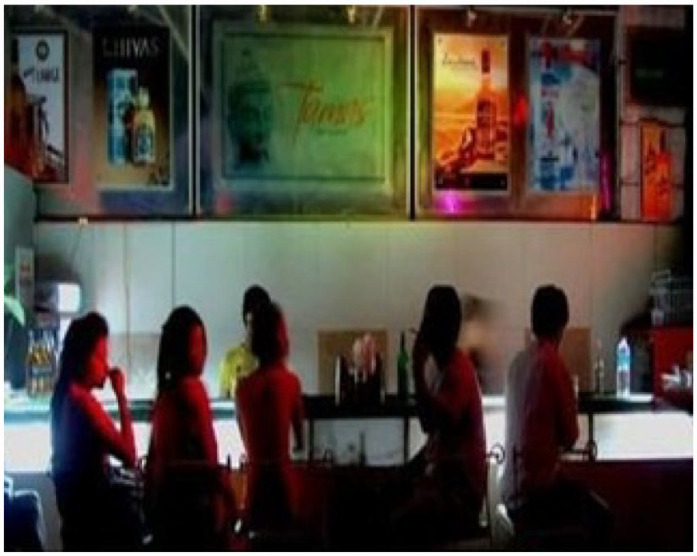
Harmful Drinker, P03, female aged 44 Image of people drinking and smoking at a bar.

**Photo 4:**
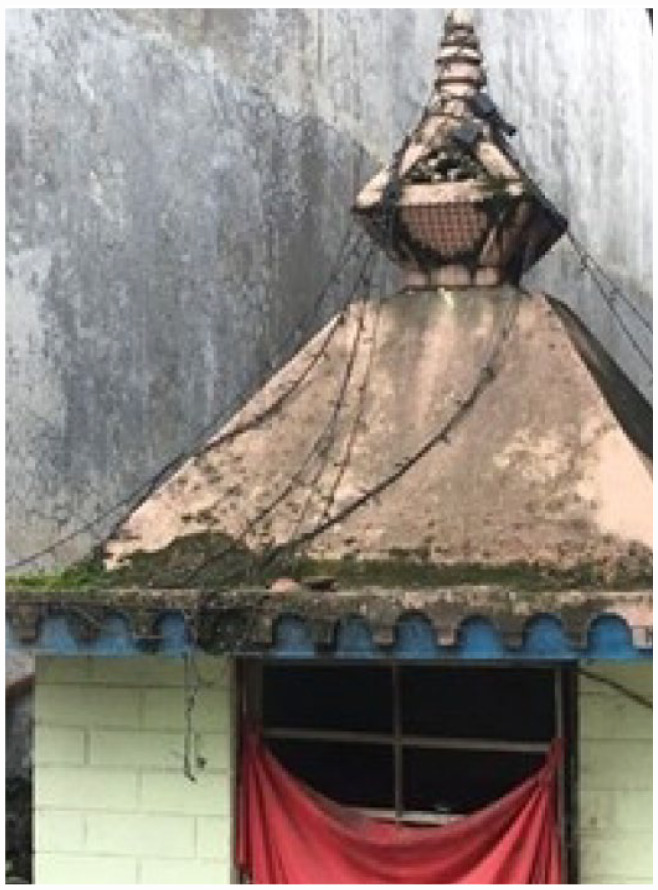
Harmful Drinker, P06, male aged 39 Image of a temple with a golden coloured pitched roof.

**Photo 5:**
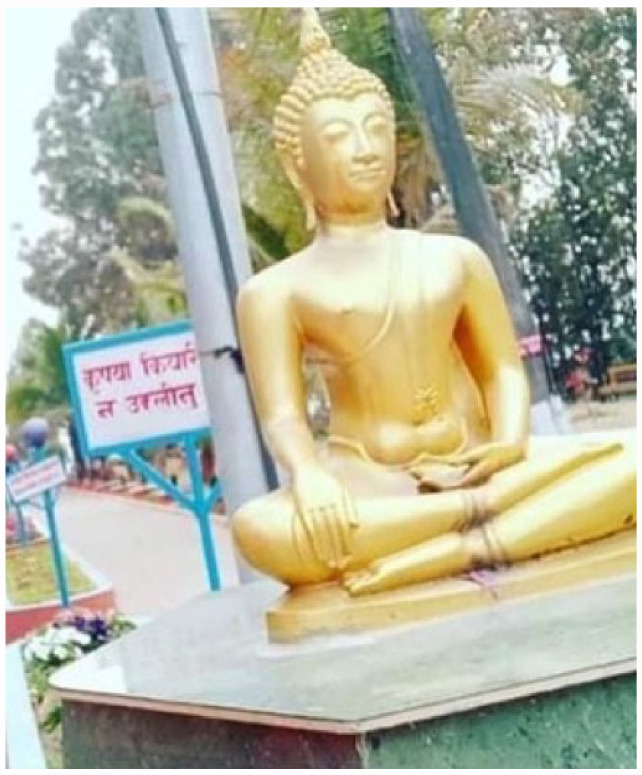
Harmful Drinker, P02, male aged 46 Image of a golden statue of Buddha seated on a platform.

**Photo 6:**
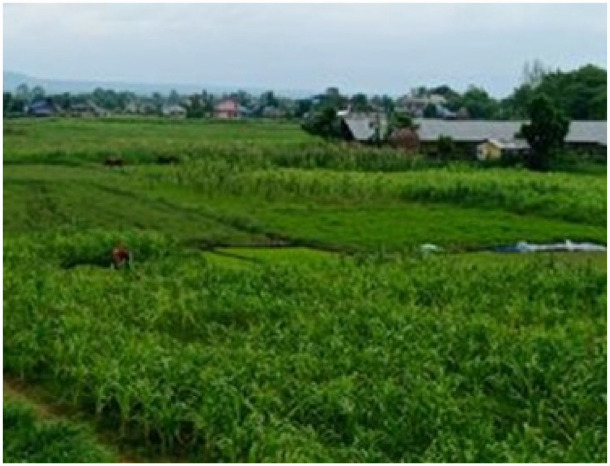
Health Worker, P05, female aged 41 Image of green farmland with a few houses and hills in the distance.

**Photo 7:**
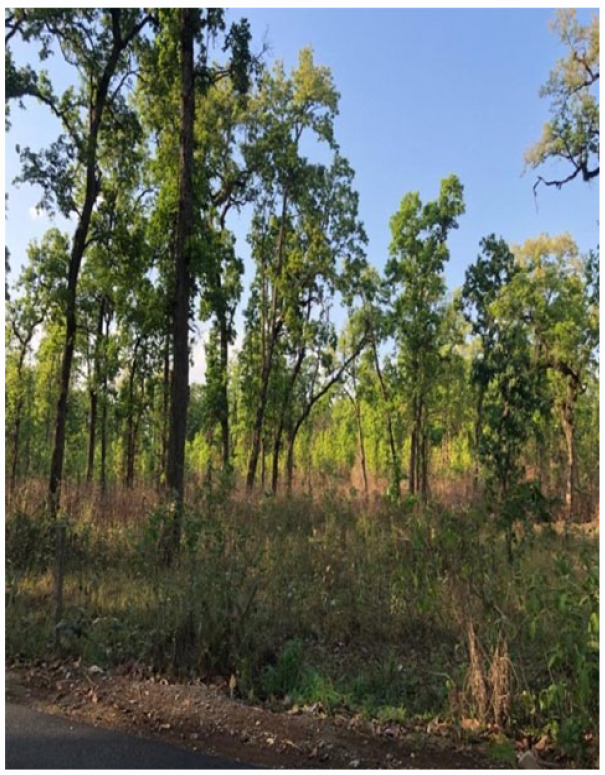
Health Worker, P02, male aged 44 Image of a forest with tall trees and low growing shrubs.

Three themes were produced following the data analysis of the photovoice interviews: ‘influences and impact of families and communities’; ‘culture and spirituality’; and ‘nature and the environment’. These themes were developed from sub-themes, which described their multifaceted nature. Presented here are each theme followed by their sub-themes (italic headings), which are illustrated with participants’ quotations and photographs (see Photos 1, 2, 3, 4, 5, 6, and 7).

### Influences and impact of families and communities

#### Strong and supportive families and communities

Participants described families and communities as strong and supportive and spoke of the interconnectedness and interdependence of these relationships. Families cared for their members, coming together for meals and festivities, encouraging individuals to seek help, and accompanying them to alcohol treatment centres. Health workers described how local alcohol services were utilised as an asset:

*For them* [alcohol users] *we provide proper counselling by helping them realise how important they are for the family and community and the nation as well . . . we help move them forward with the skills and knowledge to help to survive.* (Health Worker, Participant number (P2), male aged 44 years)

Nevertheless, both health workers and harmful drinkers alike spoke of how alcohol can undermine family and community networks. For this reason, participants believed that communities had an important role in prevention of alcohol-related harm, both at a public health and individual level.

#### People who can help

Participants described two forms of help that community members, and particularly women, could provide. Previously, supporting harmful drinkers had been regarded as a role to be undertaken by medical doctors and traditional healers. However, following mental health campaigns and training programmes this led to increased awareness about lay primary healthcare workers, and FCHV’s roles to reduce alcohol harm:

*There are FCHVs* [who] *try to provide the treatment service to that individual by persuading the family members. I have also found that the local area representatives held meetings to encourage such people to go to the health post.* (Harmful Drinker, P11, female aged 29)

Participants also asserted that communities should promote structural changes within community-based groups, health, and social care organisations, as well as attitudinal change to challenge local drinking cultures. Participants described how their community representatives could bring people together to discuss alcohol problems and develop solutions. Some participants described local networks such as the mothers’ groups and the FCHVs as similarly influential.

#### Families and communities are not necessarily an asset

Some male harmful drinkers felt that the women who ‘scolded them and gossiped’, perpetuated the stigma around alcohol. They, therefore, considered mothers’ groups and FCHVs unhelpful. Communities tended to regard harmful drinkers as selfish, irresponsible, untrustworthy, and as bad role models to children. Community leaders and health workers shunned drinkers, which had the effect of further isolating drinkers. Harmful drinkers described how community members would speak harshly and behave unsympathetically towards people who were intoxicated.

*. . . they* [community group] *normally say you* [alcohol user] *shouldn’t drink like this and don’t waste your time in such way . . . they only counsel. There isn’t any other help or support for those who drink alcohol.* (Harmful Drinker, P1, male aged 58)

### Culture and spirituality

#### Cultural norms and alcohol use

Participants were predominantly male, from higher castes, who described the diverse roles alcohol played within their lifestyles and religion. Within castes permitting alcohol use, alcohol tended to be readily available at family and community events. Participants could drink at social, cultural, and religious celebrations held at ‘party palaces’ (function halls), picnic sites, and other venues.

However, for castes which discouraged/prohibited alcohol use, harmful drinkers either brewed their own alcohol or drank in restaurants with friends. Participants concurred that, despite the deterrent effects of social censure, this pattern of drinking was causing the greatest harm to families and communities. Participants cited the benefits of exchanging knowledge between communities, and the increase in alcohol awareness provided by public health initiatives:

*In the old days people used to hide and drink. Due to hiding sometime people used to drink fast, but this* [practice] *is gradually decreasing due to greater acceptance of them having a little bit of alcohol with the family member in our community . . . People shouldn’t run or hide because of drinking alcohol but should drink in moderation.* (Health Worker, P1, male aged 48)

#### Cultural and religious resources to help harmful drinkers

Participants described the role of spirituality and faith practices in reducing alcohol harm. For some male harmful drinkers, spending time at religious sites was helpful because alcohol was prohibited there. For men and women, religious observances such as meditation, yoga, and singing hymns, provided a sense of meaning or purpose which could reduce their desire for alcohol and motivated them to reduce their drinking. Religious organisations often played an important social and charitable role in bringing men together, enabling harmful drinkers to contribute more positively to their communities. Female participants also found spirituality, meditation, and faith observances beneficial, but they tended to practice these at home rather than at religious sites.

Some health workers described meditation and yoga as an effective form of treatment for alcohol problems, and as a long-term alternative to prescribed medication. Several participants advocated an increased emphasis on yoga within primary care services.

*I have clicked the pictures of the temples and organisations . . . worshipping the gods and goddess is our belief . . . religious organisations can encourage a person to save the money used for buying alcohol so that in the future he could use that money for other purpose* (Harmful Drinker, P6, male aged 39)

### Nature and the environment

#### Agriculture and gardening as livelihood and leisure

A third of participants were farmers and described how they enjoyed gardening and horticulture as leisure activities. Many harmful drinkers described agriculture and gardening as an activity which distracted them from thoughts about drinking:

*I have not taken any treatment services instead of that I control myself while drinking . . . I try to control my drinking habit by making myself busy working in the farm . . . Being busy in the farm I don’t have time to think about gathering with friends and getting drunk*’. (Harmful Drinker, P10, female aged 33)

Among both harmful drinkers and health workers, this was a theme that female participants particularly emphasised.

#### The natural environment as a social and spiritual space

Chitwan is renowned for its natural beauty. Participants described how they enjoyed socialising with friends and colleagues at picnic sites and swimming spots. Several harmful drinkers described how people drank less at open-air social gatherings than in restaurants, because they could derive camaraderie and pleasure from the scenery and wildlife rather than from alcohol alone.

For many participants, the outdoor environment provided not only livelihood, leisure, or social opportunities, but also spiritual or personal experiences. Participants described being among natural beauty as calming and uplifting, and effective in reducing preoccupation with or craving for alcohol:

*. . . garden, parks also help in the reduction of the alcohol . . . while clicking the picture of the flowers and garden I forget all the pain, stress as well as tension . . . Sitting in the garden and looking the scenery can help to release the stress . . . and can dwell the mind in the surrounding rather than in the alcohol.* (Health Worker, P5, female aged 41)

### Asset mapping findings

**Figure 2:**
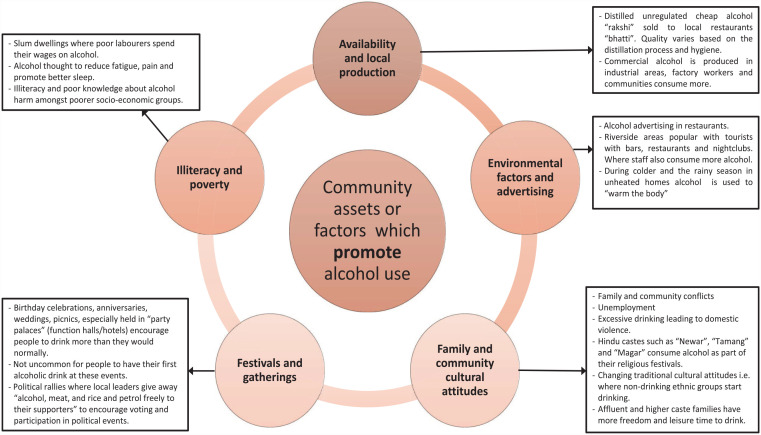
Cultural and community assets and factors, which promote alcohol consumption

**Figure 3:**
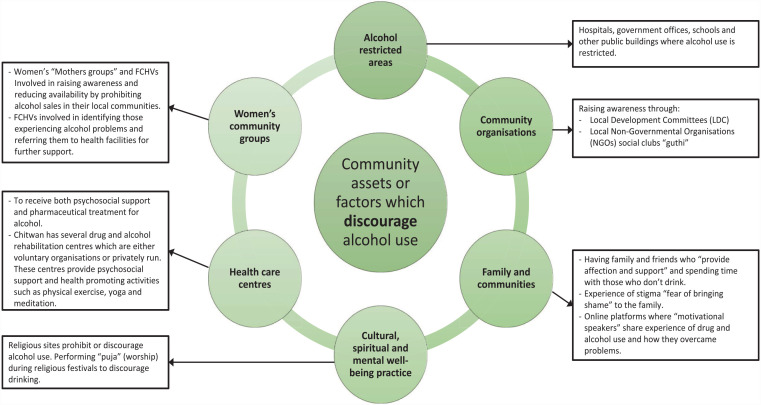
Cultural and community assets and factors, which discourage alcohol consumption

A total of five community assets considered to promote alcohol use along with six assets likely to discourage consumption were produced through the analysis and are presented in Figures 2 and 3. Those from certain ethnic groups consumed more alcohol than others, experienced family discord or felt stigmatised due to their drinking. Assets and factors relating to ‘Family and communities’ were perceived to both promote and reduce alcohol use. However, having access to a supportive family, community, and faith groups were thought to reduce consumption.

## Discussion

Resources and factors, which either promoted or discouraged alcohol use were identified from the participatory asset mapping study. The photovoice interviews produced three themes, which signified the importance of families and communities; culture and spirituality; and nature and environment in shaping participants’ attitudes to alcohol.

This study identified that families and communities in Chitwan, Nepal, play a particularly significant role in preventing and treating alcohol-related harm. Belonging to a cohesive nuclear or extended family, which can offer strong kinship and compassionate alcohol support, is an important factor to build locally developed interventions on. Having a better understanding of cultural family practices and how this may differ from the often individualised philosophy of health care found in western-focussed literature would need to be examined, especially to create contextually appropriate alcohol interventions for Nepal.^32^ However, this study also identified unsympathetic and punitive attitudes towards alcohol, which could marginalise and isolate drinkers. This supports literature exploring the relationship between social exclusion and poor outcomes following alcohol treatment.^33^

Culture, spirituality, and religion were identified as key assets to reduce drinking, as captured in participants’ photographs and the asset mapping. Nevertheless, Nepal is a nation of vast cultural, social, and ethnic diversity and a complex caste system; given the myriad ways that spirituality, and religion are known to interrelate, it is important to emphasise that this study’s findings cannot be assumed as generalisable across Nepali communities. Importantly, alcohol production and consumption practices vary widely across different ethnic groups, and for those seeking to utilise these findings to develop alcohol interventions, cultural competence and cultural humility will remain important. This study found that local religious practices were a particularly strong motivation to reduce drinking, particularly for men. This study adds a Nepali perspective to the literature.

Engaging with the natural environment is beneficial to individuals who experience substance use or mental health problems, particularly to reduce anxiety, depressive, and stress-related disorders.^34^ This study found nature and the environment to be valuable assets to drinkers and health workers alike. This was captured through nearly all participants’ photographs as well as their accounts of the intersections between the natural environment and their local culture, lifestyles, and worldviews.

### The public health concept of alcohol use

The study found Nepali men and women had different patterns of drinking, chose different places to drink and accessed community resources differently. Nepali women have traditionally been known not to drink alcohol or consume less than men.^35–37^ It remains unclear how accurate these perceptions are and whether drinking behaviour may be changing. Because of the ethnic and cultural diversity of the country, the general pattern of alcohol use presented in Figure 1 may not be an accurate reflection of alcohol consumption.

When participants described alcohol harm, they often referred to more serious consequences of drinking, often related to alcohol dependence syndromes which are likely to require medical treatment in healthcare facilities. Participants appeared to hold a professionalised understanding of alcohol support, whether medical or primary care practitioners or traditional healers. For example, managing alcohol use was considered the realm of experts, rather than something lay people could do for themselves. Our study identified that while drinkers and communities saw women as care giving figures, exemplified by FCHVs, primary health care workers or medically trained personnel were regarded as more credible and effective.

### Using photovoice interviews to explore drinking in Nepal

In recent years, photovoice and its adapted forms have been used in numerous qualitative health studies in Nepal^38,39^ and elsewhere.^40^ This study may be the first to use photovoice approaches in alcohol research in Nepal. Photovoice approach is likely to have enabled participants to speak openly about their experiences and relationships with alcohol, drawing on photos that were emotionally arousing and salient for them. It supported participants to feel in control about what to photograph and which images to share with the researcher.

### Implications for future research

In cultural and community settings such as Chitwan, our findings suggested the amplified impacts of being shunned by own families and communities, leading to social isolation and reduced access to support. Further research is needed to examine perspectives of other community members, especially women, as harm to others through alcohol use, especially against women, children, and other forms of gender inequality is a global concern.^41^ Future research should consider factors such as: availability of alcohol; alcohol advertising; negative attitudes towards alcohol users; festivals and gatherings; and illiteracy and poverty, which promote consumption. As well as examining how resources and factors such as: legislation on restricting alcohol use; community organisations such as Non-Governmental Organisations (NGOs); spiritual sites; healthcare facilities; and women’s community groups discourage alcohol use for future work.

There is limited research exploring how spirituality might prove to be an asset to reduce alcohol harm.^42^ Such research is urgently needed, given our results on the motivating and supporting role of spirituality, with religious venues and faith group members seen as sources of support for drinkers. Future alcohol interventions research should also explore and optimise assets embedded in nature and the environment. To map the alcohol consumption levels and patterns among an ethnically and culturally diverse Nepali population, a more detailed prevalence survey is needed. Finally, to translate our findings from the asset mapping and photovoice into alcohol interventions tailored to the local needs and assets, participatory workshops using the Experience-Based Co-Design (EBCD) methodologies^17^ pulling key stakeholders including the drinkers in the wider community together are the natural next steps.

### Challenges, limitations, and strengths

This study was undertaken during the height of Nepal’s COVID-19 pandemic, which created methodological challenges. This restricted travel and prevented Nepali and UK researchers from collaborating as closely as planned. The study was conducted during the monsoon season and the heavy rains caused additional barriers to communication and project activities.

The limitations of this research were the gender imbalances among participants. The cultural context and subject matter may have discouraged some from participating. Participants were disproportionately male; therefore, findings represented the perspectives of mainly male drinkers and health workers. Nevertheless, the two study components complemented one another instructively, both in identifying cultural and community resources and in understanding how people view and utilise these resources. Consequently, the research gained insight of how gender, family, and caste dynamics are embedded to determine engagement with these resources, and how some resources could either be an asset or a liability or both, depending upon the context.

The photovoice interview method may have important implications to better understand the challenges faced by those who experience alcohol harm and possibly reduce the stigma of talking about alcohol.^43^ Viewing participants’ photographs also aided discussion between Nepali and UK researchers, stimulating discussion within the team and proving useful when clarifying misunderstandings caused by translation of the interviews. Nevertheless, this study has opened up possibilities for exploring other arts-based methods and facilitating culturally engaging experiences for Nepali participants and researchers alike.^44^

The photovoice interview proved to be an effective method, which produced three themes signifying the importance of families and communities; culture and spirituality; and nature and environment as important assets and factors in shaping participants’ attitudes to alcohol. In addition, study participants were harmful drinkers or health workers; thus, findings reported were context specific and expressed by those with lived experience of alcohol, including the effects of others’ alcohol use or were involved in providing health care support to their communities. Therefore, these factors added to the strength of the study findings.

## Conclusion

This study provided new insights of how cultural and community assets, considered by local communities in Chitwan, Nepal, could both promote and reduce alcohol use, and that these should be explored within their situational context. The study identified new potential to build on visual participatory methods for future research and explore other arts-based enquiry to co-design inclusive and culturally engaging alcohol interventions.
